# Case Report: MRI Diagnosis of Wilson's Disease in a 3‐Year‐Old Dalmatian

**DOI:** 10.1111/vru.70087

**Published:** 2025-09-25

**Authors:** Natalie Durant, Matthew Paek, Wilfried Mai

**Affiliations:** ^1^ Department of Clinical Sciences and Advanced Medicine School of Veterinary Medicine University of Pennsylvania Philadelphia Pennsylvania USA; ^2^ VetRad Worthington Ohio USA

**Keywords:** brain metabolic disorders | copper hepatopathy | dog

## Abstract

A 3‐year‐old Dalmatian was presented with anorexia, vomiting, and lethargy that progressed to neurological signs with a mixed hepatopathy. MRI identified bilaterally symmetric, ill‐defined hyperintensities in the thalamus, medial and lateral geniculate bodies, and red nuclei on T2‐weighted (T2W) and transverse T2W fluid‐attenuation inversion‐recovery (T2‐FLAIR) images, and bilaterally symmetric, ill‐defined T1‐hyperintensities in the lentiform nuclei and thalamus suggestive of an underlying metabolic dysfunction. Systemic workup revealed an underlying hepatopathy. A hepatic biopsy revealed severe copper‐associated hepatitis with a digital copper quantification of 3052 µg/g dry weight. Clinical signs and MRI changes both improved following chelation therapy. The MRI findings and hepatic biopsy results led to a diagnosis of copper storage hepatopathy, consistent with Wilson's disease. This is the first description of brain MRI findings secondary to Wilson's disease in dogs.

## 1 Signalment, History, and Clinical Signs

A 3‐year‐old male castrated Dalmatian was presented to his primary veterinarian for a history of occasional vomiting, intermittent anorexia, lethargy, depression, and discomfort. Hematological and serum biochemical findings at this time included elevated ALP 534 U/L (reference range 20–150 U/L), ALT 859 U/L (reference range 10–118 U/L), a mild leukocytosis of 17.9 × 10^3^/µL (reference range 6–17 × 10^3^/µL), and mature neutrophilia of 15.38 × 10^3^/µL (reference range 3–12 × 10^3^/µL). Abdominal radiographs revealed a gas distension of the stomach. Concern for a foreign‐body obstruction at this time prompted referral for further diagnostics.

Abdominal ultrasound did not show a foreign body. The liver had a mottled parenchyma but was otherwise normal in size. The gallbladder and biliary tract were unremarkable. Small (2–3 mm) cystoliths were present in the urinary bladder. The patient was discharged at this time but re‐presented 2 days later because of an onset of neurological signs, including walking into walls, ataxia, and falling over. On physical examination, the patient had dull mentation with no response to auditory or visual stimuli. Other signs included tetraparesis with general proprioceptive ataxia, occasional subtle signs of vestibular ataxia, abnormal limb positioning (splayed, crossed, or abducted), and decreased paw replacement in all four limbs (mild in thoracic limbs and marked in pelvic limbs). The patient resisted lateral and dorsal recumbency during the examination. During hospitalization, the patient alternated between dull and stuporous mentation and subsequently developed bilateral mydriasis, hyperesthesia, and occasional knuckling of the thoracic limbs. Notable serum biochemical findings during hospitalization included mildly decreased BUN (5 mg/dL; reference range 7–27 mg/dL), increased ALT (713 U/L; reference range 0–125 U/L), and ALP (642 U/L; reference range 23–212 U/L). An ACTH stimulation test revealed normal baseline cortisol (4.2 µg/dL; reference range 1–5 µg/dL) and a mildly low post‐stimulation level (6.5 µg/dL; reference range 8–17 µg/dL). Ammonia level was normal (16 µmol/L [reference range 0–98 µmol/L]). Pre‐ and post‐prandial bile acids were elevated (Pre: 149.5 µmol/L, reference range: 0–6.9 µmol/L; Post: 280.6 µmol/L, reference range: 0–14.9 µmol/L). The patient was started on prednisone, doxycycline, trimethoprim sulfate, *S*‐adenosylmethionine, and glutathione supplementation. Following treatment, the patient's ALT and ALP decreased but remained elevated (ALT 558 U/L, ALP 419 U/L).

After 4 days of hospitalization, the patient was referred to a neurology specialty center. Physical and neurological examination findings were static at this time. The neuroanatomical localization was suspicious for a cervical spinal cord or caudal brainstem dysfunction.

## 2 Imaging, Diagnosis, and Outcome

**FIGURE 1:**
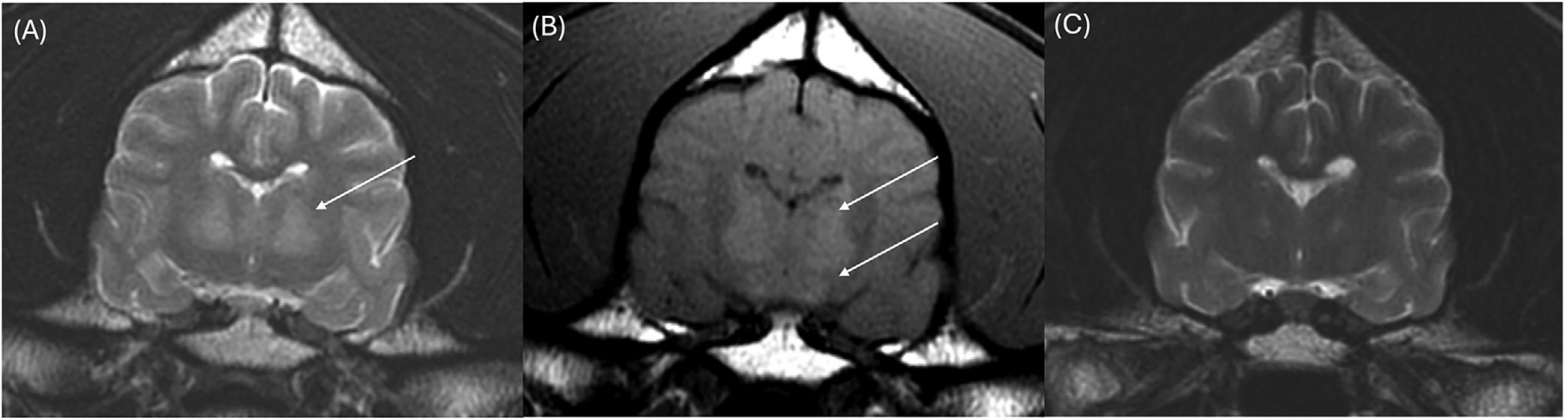
Bilaterally symmetric, ill‐defined T2 (A) and T1 (B) hyperintensities in the thalamic nuclei (white arrows) prior to copper chelation therapy; (C) markedly improved T2‐hyperintensities in the thalamus nucleus 7 months after copper chelation therapy was initiated.

**FIGURE 2:**
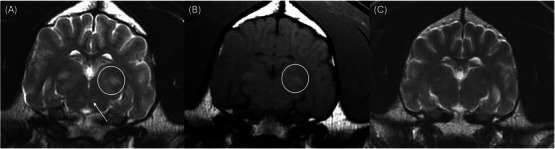
(A) Bilaterally symmetric, ill‐defined T2‐hyperintensities in the region of the medial and lateral geniculate nuclei (white circle) and red nuclei (white arrow) prior to copper chelation therapy; (B) bilaterally symmetric, ill‐defined T1‐hyperintensities in the region of the medial and lateral geniculate nuclei (white circle) prior to copper chelation therapy; (C) markedly improved T2‐hyperintensities in the region of the medial and lateral geniculate nuclei and red nuclei 7 months after copper chelation therapy was initiated.

**FIGURE 3:**
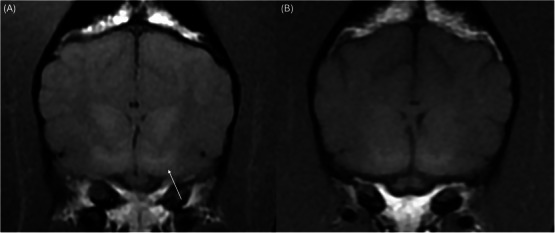
(A) Bilaterally symmetric, ill‐defined T1‐hyperintensities in the globus pallidus (white arrow); (B) transverse T1W image showing bilaterally resolved lesions in the globus pallidus 7 months after copper chelation therapy was initiated.

The patient was anesthetized for an MRI of the brain using a 1.5 T MRI scanner (Signa Genesis, GE Healthcare). The image series of the brain included transverse T2‐weighted (T2W), transverse T2W fluid‐attenuation inversion‐recovery (T2‐FLAIR), transverse T2*‐weighted fast field echo, transverse T1‐weighted (T1W) pre‐contrast, and T1W post‐contrast in all three planes. An abbreviated study of the cervical spine was also obtained, including sagittal and transverse T2W series, dorsal T1W series, and sagittal half Fourier single‐shot turbo spin‐echo (HASTE). Slice thickness varied between 3 and 5 mm. Images of the cervical spine were mostly unremarkable, only showing mild non‐compressive protrusion of the annulus fibrosus of the cervical intervertebral discs. Brain imaging identified bilaterally symmetric, ill‐defined T2 and T2‐FLAIR hyperintensities affecting the thalami (Figure 1A), medial and lateral geniculate bodies (Figure 2A), periaqueductal gray matter, and red nuclei (Figure 2A). There was T1‐hyperintensity within the lentiform nuclei bilaterally (Figure 3A), the thalami (Figure 1B), and geniculate nuclei (Figure 2B). These lesions did not display contrast enhancement. The remainder of the MRI study showed no abnormalities in any visible osseous or soft tissue structures. The findings were suggestive of a metabolic, toxic, or degenerative process. Metabolic dysfunction (i.e., copper‐associated hepatopathy such as Wilson's disease) or hepatotoxin was prioritized over a neurodegenerative process (i.e., subacute necrotizing encephalomyelopathy such as Leigh syndrome) due to the suspicion of underlying hepatopathy. A cerebellomedullary cistern cerebrospinal fluid sample was collected for analysis, which was normal.

Ultrasound‐guided liver biopsies and metabolic testing were performed. Histopathologic evaluation revealed severe copper accumulation with lobular hepatitis characterized by marked, generalized, multifocal, chronic, lymphohistiocytic, centrilobular hepatitis with marked copper accumulation, central–central bridging fibrosis, mild centrilobular dissecting fibrosis, copper granulomas, centrilobular collapse, and regenerative nodules. Additionally, moderate lymphocytic lobular hepatitis with individual necrotic hepatocytes was suggestive of an immune‐mediated response (thought to be caused by sensitization against epitopes on damaged hepatocytes from the copper toxicity). Digital copper quantification testing showed markedly increased copper levels at 3052 µg/g dry weight (normal <400 µg/g). Rhodanine histochemical stains revealed severe hepatocellular copper accumulation (rhodanine score 4 out of 5). The patient was diagnosed with Wilson's disease and was started on copper chelation therapy (d‐penicillamine), phosphatidylcholine, and a protein‐restricted diet.

Approximately 7 months after initiating copper chelation therapy, the patient presented for a neurology recheck appointment. At this time, the patient was doing well at home with a resolution of all clinical signs. Physical and neurological examinations were normal, and liver enzyme values had returned to normal levels. A repeat MRI of the brain was performed with the addition of a T2W dorsal plane sequence to replicate an axial T2W sequence used in humans. Compared to the pretreatment study, there was marked improvement in all the previously noted T2‐ and T2‐FLAIR hyperintensities. There were very mild bilaterally symmetric, ill‐defined T2‐hyperintensities in the thalamus (Figure 1C), medial and lateral geniculate bodies, and red nucleus (Figure 2C). The previously noted regions of T1 hyperintensities in the lentiform nucleus had resolved (Figure 3B).

## 3 Discussion

Wilson's disease is rare in humans. It is an autosomal recessive condition caused by a mutation in the ATP7B gene, which results in copper transport errors and causes copper accumulation in the liver and deposition in other sites, notably the brain [1, 2]. In people, clinical signs can range from asymptomatic to severe and cause hepatic, neurologic, and psychiatric symptoms [2]. Wilson's disease can cause asymptomatic liver enzyme elevation, acute liver failure, acute‐on‐chronic liver failure, and cirrhosis [2]. Clinical manifestations of neurologic symptoms in humans are secondary to nerve cell damage resulting from copper deposition, causing edema, necrosis, and spongiform degeneration [3]. Common neurologic manifestations are extrapyramidal symptoms, including dysarthria, dystonia, Parkinson's disease symptoms (rigidity, resting tremor, and bradykinesia), cerebellar disturbances, and choreoathetosis [2, 4, 5]. A variety of psychiatric abnormalities can be seen in people with Wilson's disease, which can be present before the onset of hepatic and other neurological signs [2]. In humans, the diagnosis of Wilson's disease is established on serum ceruloplasmin levels, copper levels (urinary excretion, serum, and hepatic), and the presence of Kayser–Fleischer rings on ophthalmologic examination [1, 2].

In dogs, hepatic copper toxicosis is a well‐recognized entity. It can result from a primary metabolic defect in hepatic copper metabolism, like Wilson's disease in humans, or from an altered hepatic biliary excretion of copper. Inherited copper‐associated hepatopathies have been documented in various breeds, such as the Bedlington terriers. Copper‐associated liver disease is also described in Dalmatians [6], though neurologic signs have not been documented thus far.

In this dog, a diagnosis of Wilson's disease was made on the basis of MRI findings and the presence of copper‐associated hepatopathy on liver biopsies. It was supported by the positive clinical response to chelation therapy.

In this case, hepatic encephalopathy was an important differential based on the clinical presentation. Hepatic encephalopathy is most often seen in dogs with congenital vascular anomalies such as portosystemic shunts but can also occur in hepatopathies that cause portal hypertension or acute fulminant hepatic failure [7]. In this patient, clinical signs were first noted only 3 weeks prior to the onset of neurological signs. Due to the rapid disease progression and unknown etiology at the time, acute fulminant hepatic failure was a concern in this patient. MRI features of hepatic encephalopathy include brain atrophy, bilateral symmetrical T2‐, and T2‐FLAIR hyperintense lesions affecting the white and gray matter equally, and bilateral symmetric T1‐hyperintensity in the lentiform nuclei [8]. This patient showed no signs of brain atrophy or white matter lesions on the pretreatment MRI study. In addition, hyperammonemia is a central mediator in the pathophysiology of hepatic encephalopathy and was not present in this dog.

MRI findings of Wilson's disease have been described in the human literature. They are caused by both cerebral copper accumulation and secondary nerve cell damage, including edema, necrosis, and spongiform degeneration [3]. Symmetric bilateral deep gray matter signal alteration, commonly seen as concentric laminar hyperintensities, is often seen on T2W and T2‐FLAIR images [1, 3]. Cerebral structures commonly affected in humans with Wilson's disease include the lentiform nuclei, thalami, caudate nuclei, midbrain, and pons [1, 3]. Abnormal bilateral increased signal intensities in the basal ganglia on both T2W and T1W images are an important finding of Wilson's disease [1, 5]. This patient did display bilateral T2‐ and subtle T1‐hyperintensities in the thalami, but only T1‐hyperintensity of the lentiform nucleus. T1‐hyperintensity in the lentiform nuclei has been reported in dogs with hepatic encephalopathy due to manganese accumulation in the globus pallidus secondary to decreased hepatobiliary excretion [9]. This is reversible when normal hepatic function is restored [10]. Copper accumulation is also a cause of high T1 signal on MRI and is likely the origin of the changes observed in this dog [11].

The “face of the giant panda” sign is a characteristic MRI feature of Wilson's disease in humans, seen on axial plane T2W images. It is, however, only seen in a minority of patients [1, 3]. This sign results from the selective contrast caused by hyperintensity in the midbrain with sparing of the red nuclei, the lateral portion of the substantia nigra, and superior colliculi on axial T2W images [1, 3]. A dorsal imaging plane in veterinary patients is the closest representation of an axial imaging plane in humans and presumptively where the “face of the giant panda” sign may be seen. In this patient, a dorsal T2W sequence was not performed in the pre‐chelation study, and the “face of the giant panda” sign was not seen on the dorsal T2W sequence in the post‐chelation study; however, there was significant improvement in the MRI changes on T2W images at that time. In addition, this patient had bilateral T2‐hyperintense signals in the red nuclei; therefore, it is unlikely that this MRI sign would have been present at the time of the initial MRI. This may represent a species‐specific difference in the presentation of Wilson's disease on MRI [12]. With copper chelation treatment, Wilson's disease lesions can regress [1], as was observed in this patient on the post‐chelation MRI.

In conclusion, this case describes unique MRI findings in a young dog with severe copper‐associated hepatopathy (Wilson's disease), which, to the authors’ knowledge, have not previously been reported in the veterinary literature. It documents clinical and MRI improvement after copper chelation therapy. Wilson's disease should be considered as a differential in dogs with hepatopathy (of unknown etiology or known copper‐associated hepatitis), neurological signs, and bilaterally symmetric deep gray matter lesions on MRI.

## Author Contributions

**Category 1**

(a) Conception and Design: Lehman, Pierce
(b) Acquisition of Data: Lehman, Pierce
(c) Analysis and Interpretation of Data: Lehman, Saboda, Love, Pierce

**Category 2**

(a) Drafting the Article: Lehman, Saboda, Love, Pierce
(b) Reviewing Article for Intellectual Content: Lehman, Saboda, Love, Pierce

**Category 3**

(a) Final Approval of the Completed Article: Lehman, Saboda, Love, Pierce

**Category 4**

(a) Agreement to be accountable for all aspects of the work in ensuring that questions related to the accuracy or integrity of any part of the work are appropriately investigated and resolved: Lehman, Saboda, Love, Pierce

## Disclosure

The findings presented herein were not presented at scientific meetings or published in abstracts. The study adhered to EQUATOR Network CARE guidelines.

## Ethics Statement

The use of de‐identified patient information was authorized by the Hospital Director. All diagnostic procedures, treatments, and interventions described in this case report were performed in accordance with established veterinary ethical guidelines. Informed consent was obtained from the animal's owner for the diagnostic imaging procedures and treatments, and the case details were included in this report. The patient's welfare was prioritized at all times, and the interventions described were undertaken with the primary aim of improving the patient's health and well‐being.

## Conflicts of Interest

The authors declare no conflicts of interest.

## Data Availability

Data sharing is not applicable to this article, as no new data were created or analyzed in this study.
